# Solitary Scalp Metastases of Parotid Pleomorphic Adenoma: A Case Report

**DOI:** 10.7759/cureus.33723

**Published:** 2023-01-12

**Authors:** Raghad Wasil AlNahwe, Abdullah Abdulaziz Al Ghamdi, Ziyad Mosleh Alanazi, Lama AlSahli, Razan Aldhahri, Alaeddin Jebreel

**Affiliations:** 1 College of Medicine, Royal College of Surgeons in Ireland, Dublin, IRL; 2 College of Medicine, King Saud Bin Abdulaziz University for Health Sciences, Riyadh, SAU; 3 Otolaryngology - Head and Neck Surgery, King Faisal Specialist Hospital and Research Centre, Riyadh, SAU

**Keywords:** a case report, parotid gland metastasis, solitary metastasis, scalp lesions, pleomorphic adenoma of parotid gland, salivary gland tumor, salivary gland neoplasm, benign pleomorphic adenoma

## Abstract

Pleomorphic adenoma (PA) of the parotid gland is considered one of the commonest benign salivary gland neoplasms in both adults and pediatrics. However, metastatic pleomorphic adenoma (MPA) is extremely rare. In the past three decades, multiple cases were reported in the literature of MPA, where the metastatic phase has been preceded by a local recurrence for the majority of the cases. Metastases to the lungs, liver, bone, head and neck were reported. This paper will discuss a rare case presentation of MPA that metastasised solely to the face and scalp subcutaneous tissue with no other sites of metastases in a male adult.

## Introduction

Salivary glands are divided into major and minor glands. The major glands are parotid, submandibular, and sublingual glands. Tumours of the salivary glands are considered rare entities as they only constitute 3-4% of all head and neck tumours [1]. The latest research in the literature showed that 80% of the tumours were benign, and the majority originated from the parotid gland [2,3]. A wide histological variety has been identified by the World Health Organization [4]. Pleomorphic adenoma (PA), also known as benign mixed tumour, is the commonest type of benign tumour across all salivary glands [2]. However, a retrospective study that was done in northern Greece suggested that PA comes second to Warthin’s tumour [5]. PA can transform into malignant carcinomas as well as metastasise [6]. Although metastases are uncommon, it has been reported in the literature that when a PA metastasises, also known as metastatic pleomorphic adenoma (MPA), it would still exhibit its benign behaviour distally [7]. The World Health Organization classified it as a malignant neoplasm because of its capacity to metastasise and locally recur after excision [4]. The most common sites of metastases are bones, lungs, and lymph nodes [6,8]. In this paper, we would like to present a case of metastatic pleomorphic adenoma that presented with multiple skin lesions. Such cases were scarcely reported in the literature based on our literature review.

## Case presentation

**Figure 1:**
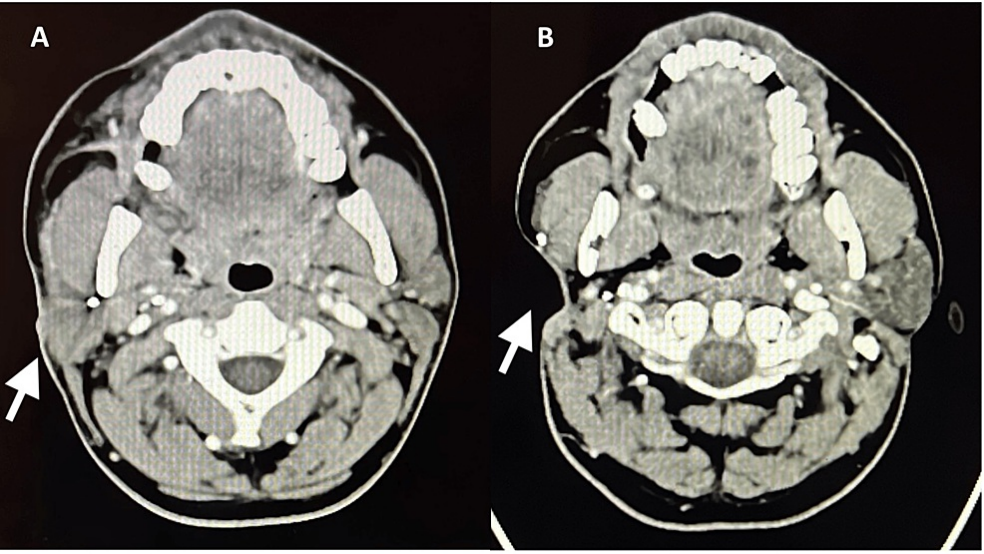
(A) CT scan, Arrow: shows right-sided parotid local recurrence. (B) CT scan, Arrow: area post revision parotidectomy.

**Figure 2:**
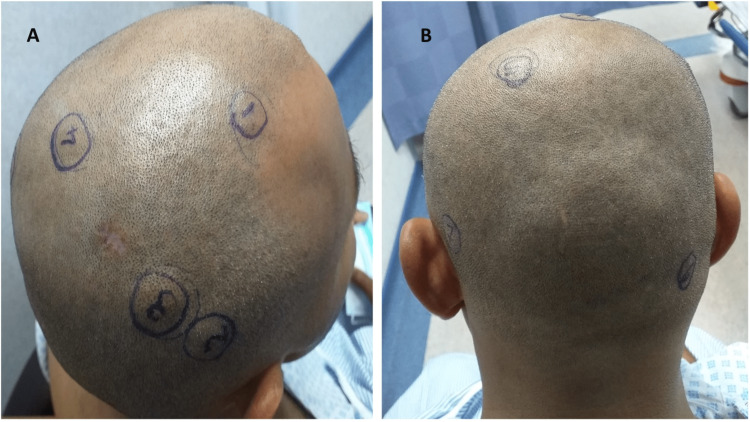
(A) and (B) Seven scalp lesions identified on histopathology as pleomorphic adenoma.

A 40-year-old male presented to the clinic at the age of 25 with a right-sided parotid swelling progressing over a few months. Fine needle aspiration was done and the results came back positive for pleomorphic adenoma, for which he underwent a right-sided parotidectomy in 2007. With the initial presentation, the patient had no lymph node involvement both clinically and radiologically. Ten years post-surgery, he developed local recurrence and underwent revision parotidectomy as shown in the CT scan in Figures 1A, 1B. Both surgeries had negative margins according to the histopathology report, and the facial nerve was spared. Accordingly, the patient had no facial nerve palsy. It was reported as 'Recurrent multifocal pleomorphic adenoma, negative level II lymphnodes, no evidence of malignancy, there's fibrosis and traumatic neuroma due to previous surgical intervention'. Furthermore, to ensure the complete eradication of the recurrent PA, the patient received adjuvant radiotherapy as part of his treatment plan. In 2019, the patient represented to the clinic with a right eyebrow subcutaneous lesion measuring 1.1 𝗑 0.6 𝗑 0.5cm, which was excised and showed the same histopathological picture of PA. This was the first recorded metastasis in this case after 12 years of initial diagnosis and two years after the recurrence. Thereafter, the patient started developing multiple scalp lesions. Seven scalp lesions were identified in this patient’s case, as shown in Figures 2A, 2B, ranging from 0.6 cm to 0.9 cm, and were biopsied under general anesthesia. Histopathology reported the lesions as PA with no malignant features and only one of the seven lesions exhibited lymphovascular invasion. Thus, a diagnosis of metastatic pleomorphic adenoma was made. 

## Discussion

In a recent case review study, over 5000 cases of salivary gland tumours were reviewed over a period of almost 40 years [3]. The authors concluded that the parotid gland had the highest incidence of both benign and malignant neoplasms across all age groups. Furthermore, the majority of the neoplasms were PA, accounting for 68%.

The literature has established that PA exhibits the capability of recurrence at the primary site or metastasising to a secondary site [6,8,9]. Both of the aforementioned possibilities developed in our case. A systematic review showed that the most common sites for metastasis would be bones, lungs, and neck lymph nodes, respectively [10]. Very occasionally, however, PA metastases have been identified in the liver and kidneys [11,12]. On the same notion, parotid gland malignant tumours, such as acinic cell carcinoma, were also reported to metastasise to unusual sites, including the abdominal wall, cavernous sinus, and sternum [13,14].

In reference to our case, PA originating from the parotid glands that metastasise to the scalp is virtually not encountered as often. After a thorough literature review, we concluded that the first and only case report of such a clinical scenario was described by Tarsitano et al. back in 2014 [15]. The paper reported multiple PAs originating from the parotid glands that metastasised to the scalp, nasal cavity, and brain, metachronously. Similarly, our patient presented with multiple metastatic lesions that were exclusively limited to the scalp and face. Accordingly, our case is a rare presentation of a new phenomenon, where PA spread solely to the subcutaneous tissue with no other distant metastasis. 

The exact pathophysiology of MPA is still not completely understood. It was hypothesised that the rationale behind it was due to surgical manipulation leading to seeding and dislodgment of PA cells, the presence of a known risk factor such as a history of irradiation, positive margins post-surgical excision, or genomic alterations with selective advantage gaining the ability to metastasise [7,8,15-17]. In our case, the disease progression followed a chronological order, one that is similar to most cases reported in the literature. By retrospectively reviewing published cases, it was concluded that most cases would have developed MPA after their primary or secondary tumour by an average time frame of seven to 18 years [8]. However, when our patient had his first metastatic lesion, he developed these scalp lesions progressively over a very short period of time, even though he received adjuvant radiotherapy shortly before then. 

For further elaboration, surgical excision is considered the cornerstone of PA treatment [18,19]. However, complete resection could be challenging due to the parotid gland's anatomical relationships and its proximity to the facial nerve and the external carotid artery. The location and size of the tumour also play a crucial role in achieving negative margins without sacrificing the facial nerve. In fact, it has been proven that even with negative margins, patients still developed metastasis, which seemed inevitable [19-21]. This issue could be addressed by employing other treatment options in conjunction with surgery. Radiotherapy and chemotherapy can be used as neoadjuvant, adjuvant, or concurrent therapies [22,23]. 

Adjuvant radiotherapy proved effective in lengthening the remission period and providing local control [24-26]. Generally, radiotherapy is indicated for advanced disease, high-grade tumours, close or positive margins, perineural or lymphovascular invasion, and recurrent and metastatic disease [27]. Therefore, our patient had two locoregional resections for the primary and secondary tumours, which were ten years apart. Subsequently, the patient received adjuvant radiotherapy to ensure maximal eradication of neoplastic cells to further reduce the risk of metastasis. In spite of having two negative margin surgeries and a course of radiotherapy, he still developed metastases within a year of treatment completion.

## Conclusions

To the best of our knowledge, this is the first reported case of scalp MPA in Saudi Arabia. Despite the fact that MPA is extremely rare, the diagnosis should be suspected in patients with a known history of PA presenting with lesions after recurrence. Although salivary gland neoplasms account only for 3-4% of head and neck tumours, the morbidity and mortality associated with them are burdening our healthcare system. Especially in tertiary and specialized hospitals, where the majority of the cases being referred would be neoplasms such as PA. The latest research showed that the majority of cases could be treated by surgery alone, however, the risk of metastasis of such tumours is still high. Some patients were treated with neoadjuvant, adjuvant, and even concurrent chemoradiotherapy. Thus, the available treatment options are not curative. Indeed, extensive research has to be done to establish newer treatment modalities. Biological agents revolutionized the management of many medical conditions across all medical specialities. Specifically, in otolaryngology, biologics have been integrated into the management of chronic rhinosinusitis, allergic rhinitis with polyposis, and head and neck benign and malignant neoplasms. Since they showed exquisite outcomes; we suspect that biologics might play an important role in treating PA in the future. 
